# Design and Development of Fluorinated and Biocide-Free Sol–Gel Based Hybrid Functional Coatings for Anti-Biofouling/Foul-Release Activity

**DOI:** 10.3390/gels8090538

**Published:** 2022-08-26

**Authors:** Silvia Sfameni, Giulia Rando, Maurilio Galletta, Ileana Ielo, Marco Brucale, Filomena De Leo, Paola Cardiano, Simone Cappello, Annamaria Visco, Valentina Trovato, Clara Urzì, Maria Rosaria Plutino

**Affiliations:** 1 Department of Engineering, University of Messina, Contrada di Dio, S. Agata, 98166 Messina, Italy; 2 Institute for the Study of Nanostructured Materials, ISMN—CNR, Palermo, c/o Department of ChiBioFarAm, University of Messina, Viale F. Stagno d’Alcontres 31, Vill. S. Agata, 98166 Messina, Italy; 3 Department of ChiBioFarAm, University of Messina, Viale F. Stagno d’Alcontres 31, Vill. S. Agata, 98166 Messina, Italy; 4 Institute for the Study of Nanostructured Materials, ISMN—CNR, Bologna, CNR Bologna Research Area, Via Piero Gobetti 101, 40129 Bologna, Italy; 5 Institute for Biological Resource and Marine Biotechnology (IRBIM)—CNR of Messina, Spianata S. Raineri 86, 98122 Messina, Italy; 6 Institute for Polymers, Composites and Biomaterials, CNR—IPCB, Via Paolo Gaifami 18, 95126 Catania, Italy; 7 Department of Engineering and Applied Sciences, University of Bergamo, Viale Marconi 5, 24044 Dalmine, Italy

**Keywords:** sol–gel technique, anti-fouling properties, fouling release activity, marine bacteria, non-biocide release, amphiphilic coating

## Abstract

Biofouling has destructive effects on shipping and leisure vessels, thus producing severe problems for marine and naval sectors due to corrosion with consequent elevated fuel consumption and higher maintenance costs. The development of anti-fouling or fouling release coatings creates deterrent surfaces that prevent the initial settlement of microorganisms. In this regard, new silica-based materials were prepared using two alkoxysilane cross-linkers containing epoxy and amine groups (i.e., 3-Glycidyloxypropyltrimethoxysilane and 3-aminopropyltriethoxysilane, respectively), in combination with two functional fluoro-silane (i.e., 3,3,3-trifluoropropyl-trimethoxysilane and glycidyl-2,2,3,3,4,4,5,5,6,6,7,7,8,8,9,9-hexadecafluorononylether) featuring well-known hydro repellent and anti-corrosion properties. As a matter of fact, the co-condensation of alkoxysilane featuring epoxide and amine ends, also mixed with two opportune long chain and short chain perfluorosilane precursors, allows getting stable amphiphilic, non-toxic, fouling release coatings. The sol–gel mixtures on coated glass slides were fully characterized by FT-IR spectroscopy, while the morphology was studied by scanning electron microscopy (SEM), and atomic force microscopy (AFM). The fouling release properties were evaluated through tests on treated glass slides in different microbial suspensions in seawater-based mediums and in seawater natural microcosms. The developed fluorinated coatings show suitable antimicrobial activities and low adhesive properties; no biocidal effects were observed for the microorganisms (bacteria).

## 1. Introduction

Biofouling, defined as the undesired accumulation given by any association of microorganisms, algae, plants, and marine animals on submerged surfaces, causes over time bio-deterioration of exposed areas of ships, boats, ports, underwater cultural heritage, with progressive loss of economic value, as well as higher costs for their maintenance, and fuel consumption in the case of vessels [1]. In this regard, it has been calculated that the development of new anti-fouling technologies would reduce the fuel consumption for navigation (between 38 and 72%), saving 60 billion dollars and avoiding the emission of about 390 million tons of greenhouse gases each year [2]. 

In an underwater environment, biological colonization, at the base of biofouling phenomena, is a relatively fast process in which microbial species [3] and the adhesion time vary significantly depending on the geographic area, especially related to environmental conditions (i.e., salinity, pH, temperature, nutrient levels, solar irradiation, etc.) [4,5].

Several research studies have already been carried out on products that prevent marine fouling, namely anti-fouling (AF, hereafter) coatings [6,7,8]. An ideal AF coating should have the following properties: durability, resistance to external/mechanical agents, easiness to apply, low cost, and non-toxicity for non-target species and marine environment [9].

**Figure 1:**
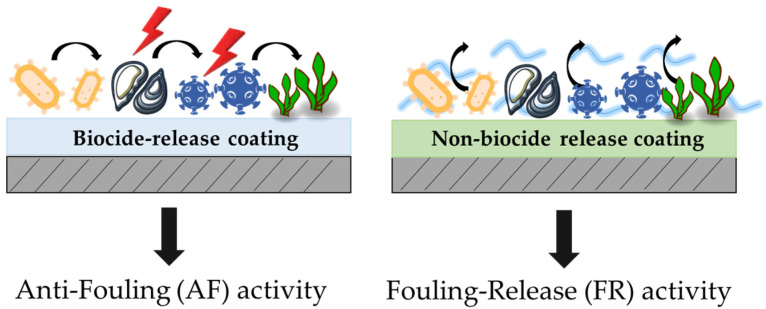
Schematization of anti-fouling and fouling release activity of sol–gel functional coatings.

The AF coatings can be classified into two categories: biocide-release coatings and non-biocide-release coatings, see Figure 1 [10,11,12]. The biocide-release coatings are based on the dispersion of biocides from different types of polymeric hosting matrices and they are progressively released over time in seawater. Currently, these coatings are the most used; however, in this regard, the problem of toxicity and high maintenance costs still remain a critical issue [13,14,15]. On the other hand, non-biocide release AF coatings represent the non-toxic and environmentally friendly alternative to anti-fouling coatings containing biocides (also called fouling release activity, FR hereafter) [16,17]. 

Two strategies are exploited in the non-biocidal approach: (i) the “separation of stabilized biofoulants”, which attempts to reduce as much as possible the force with which the micro-organisms adhere on a surface, facilitating their removal due to the weight of the deposits or by the flow of water generated during navigation; (ii) “prevention of the adhesion of biofoulants”, which aims to avoid the formation of a stable fouling film, thus preventing the adhesion of organic molecules that will trigger the bio-settlement process. Many other aspects, related to the characteristics of the materials that can be used to optimize AF or FR strategies, play an important role in controlling the non-desired biofouling process [18,19,20,21]. The chemical, physical, mechanical, and structural properties, the mass (elastic modulus, coating thickness, etc.), and topography (i.e., the physical constraints) of the covered surface are all equally important parameters, as they will determine the character of the AF coating itself and the life span of the applied material [22,23]. The coatings designed to solve these needs are usually silicones- and fluoro-polymers based on the strategy of separation of biofoulants [24,25,26]. Reticulated polyurethane/polysiloxane systems [27,28,29,30,31] and combined coatings based on fluorine and silicon demonstrate high abilities in favoring the separation of stabilized biofoulants on surfaces immersed in the marine environment [32,33,34,35,36]. 

The enormous adaptability of sol–gel methodology, which is highly controlled, provides some advantages over previous existing procedures such as low process temperatures, good homogeneity products such as low thickness coatings can be obtained, and production of mixed oxides thanks to the stoichiometric control of the composition of the starting solution, better control of the porosity of the material produced by varying the heat treatment, and a high degree of purity, but it presents also some limitations as costs of starting materials, possible formation of fractures during the cross-linking phase and long process times [37,38,39].

Methods and applications have expanded in tandem with the growing interest in sol–gel technique [40,41,42].

In this regard, sol–gel-based coatings have already been widely used to enhance the surface properties of different substrates, with which they may bind covalently and steadily, and let the final coated surface show good chemical inertness, resistance to thermal and mechanical stress, and still no cytotoxicity towards human health and environment [43,44,45]. 

As a matter of fact, currently, fluorinated long-chain derivatives are still widely employed, thanks to very low surface energy, as functional hydro repellent additives or cross-linkers to improve water repellency, together with chemical- and photo-stability properties of sol–gel-based coating, as well as coated surface. 

Hybrid organic–inorganic fluorinated materials were already prepared and used as hydrophobic coatings for conserving lithic substrates [46,47,48].

The aim of this work is to design and develop fouling release biocide-free coatings, bearing different long-chain fluorinated substituents in combination with other common sol–gel-based cross-linkers [49,50]. 

Despite the presence of nonpolar fluorinated substituents, very often (bio)fouling is still able to adhere on (super)hydrophobic coatings, leading to water penetration and subsequent coating breaking and run over [51].

Moreover, as mentioned before, hydrophobic coatings thanks to their low surface energy, present a low adhesion force towards polar marine foulants [52,53], but they are not so effective with non-polar foulants characterized by an adhesion highly related to the coating surface energy and wettability, such as some barnacle cyprids and algal zoospores [54] or other non-polar extracellular substances and cell walls components [55].

In this regard, different research studies are still devoted [56,57,58] to the design and synthesis of amphiphilic FR coatings, bearing both polar and nonpolar groups (i.e., fluoro-polymers), either also as hyperbranched or mixed cross-linked networks. With this approach, the overall amount of functional hydrophobic copolymer may be drastically reduced (<15%), since it has been shown that at the water/coating interface, the hydrophobic brushes align with each other and aggregates in a self-assembled monolayer (SAM) form [59]. Furthermore, the presence of a dynamic surface with local variations in surface chemistry, topography, and mechanical properties of such amphiphilic coatings, leads to lower interfacial interactions and therefore a less fouling settlement [60].

**Figure 2:**
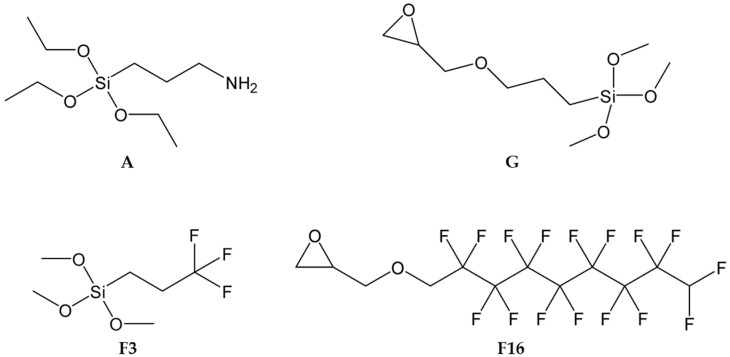
Chemical structure of the employed functional alkoxysilane sol–gel precursors together with the adopted acronyms.

Four types of silica precursors (see Figure 2) were used in order to combine the chemical-physical properties of the alkoxysilane cross-linkers subunits containing epoxy and amine groups (i.e., 3-Glycidyloxypropyltrimethoxysilane, GPTMS, and 3-aminopropyltriethoxysilane, APTES; respectively), with those of co-monomers containing functional fluorinated organic compounds (i.e., 3,3,3-trifluoropropyl-trimethoxysilane and glycidyl-2,2,3,3,4,4,5,5,6,6,7,7,8,8,9,9-hexadecafluorononylether).

Four developed (G_A, G_A_F3, G_A_F16, G_A_F3_F16) coatings are obtained by reaction of two bifunctional starting sol–gel precursors, namely APTES and GPTMS (in a concentration ratio of 1:2; hereafter also indicated as A and G, respectively, Figure 2); both of them are bearing a trialkoxysilyl group in one side, and an amine or an epoxy group, on the other, respectively. This bifunctionality of the two reacting ends of A and G favors the development of a stable sol–gel-based 3D matrix, thus guaranteeing complete and stable coverage of the treated coated surface.

Moreover, the amphiphilic coatings show to be very efficient as biocide-free FR coatings, thanks also to synergic actions coming from the polar fouling resistant a polyethylene oxide (PEO)- and polyether amine(PEA)-based, cross-linked matrix and the fouling release F3 and F16 copolymers. Finally, we thought it worthwhile to develop an asymmetric nanostructured hyperbranched polymeric coating using both F3 and F16 co-monomers, i.e., bearing both long and short perfluorinated chains.

In this way, we would rather try to simulate the lotus effect, with well-separated long chain brushes, in whose cavities (i.e., in correspondence of F3 chains) air may be entrapped, thus preventing the penetration of water and surface wettability.

All coated surfaces were characterized by different chemical–physical, morphological, and rheological techniques, and the good antibacterial and antifouling properties were assessed by the biological test. In particular, biological tests aimed, from one side, to evaluate if the coatings could have released some antibacterial compounds in the liquid medium (biocide-release), while the antifouling effect was evaluated via the reductions of the number of adhering cells on the effective surfaces as compared to the controls.

**Figure 3:**
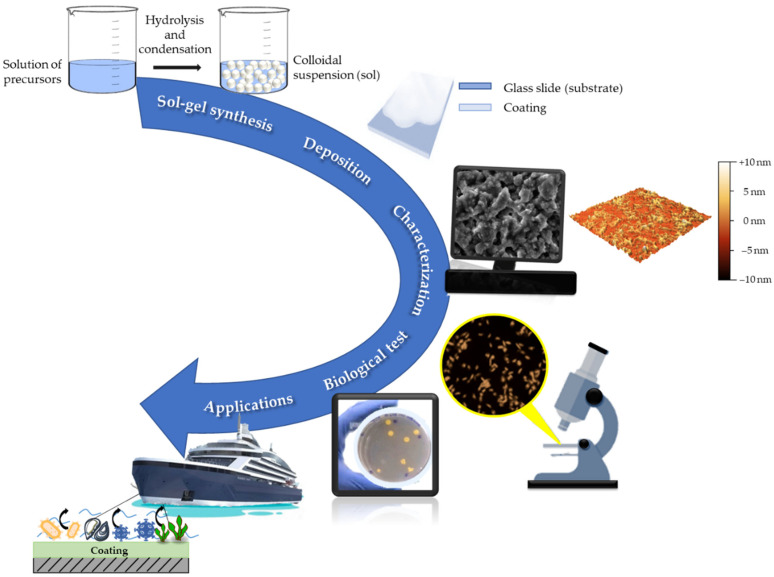
Overall aim of the work, from the FR sol–gel synthesis to blue growth applications.

These results will open the way to the development of eco-friendly, economical, durable, and easy-to-apply matrices that show also interesting fouling release and antibacterial activities, that may find useful applications in blue growth and buildings, as well as for cultural heritage protection (Figure 3).

## 2. Results and Discussion

**Figure 4:**
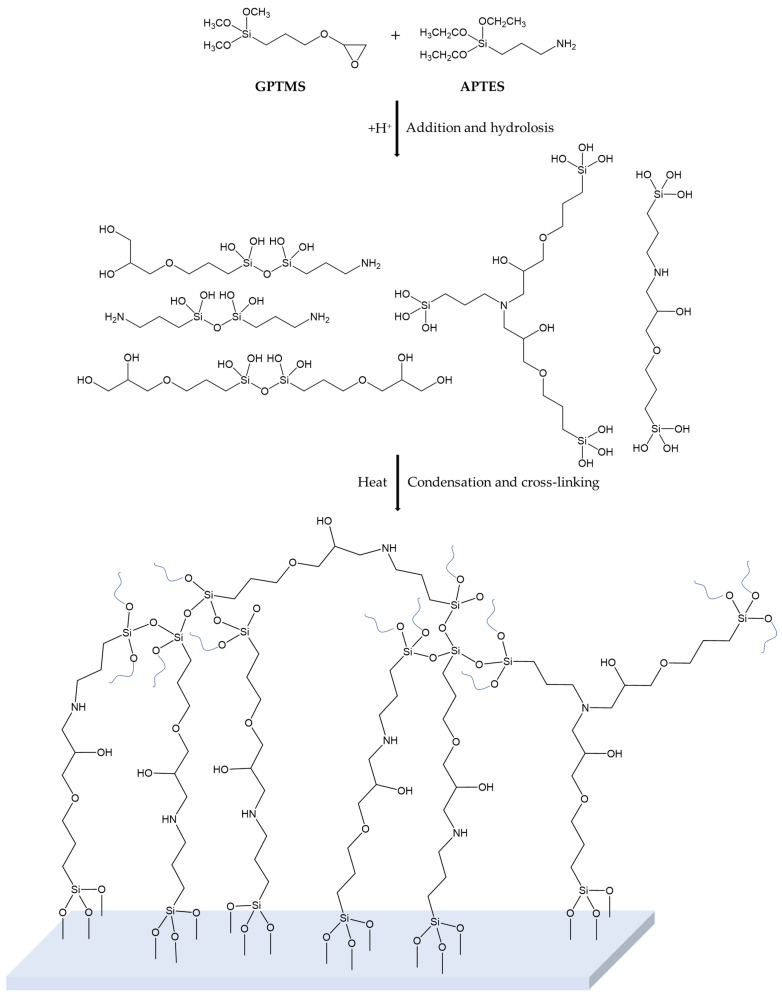
Sol–gel synthesis towards the formation of the cross-linked PEA-PEO-based G_A coating by reaction of G/A (2:1) [61].

**Figure 5:**
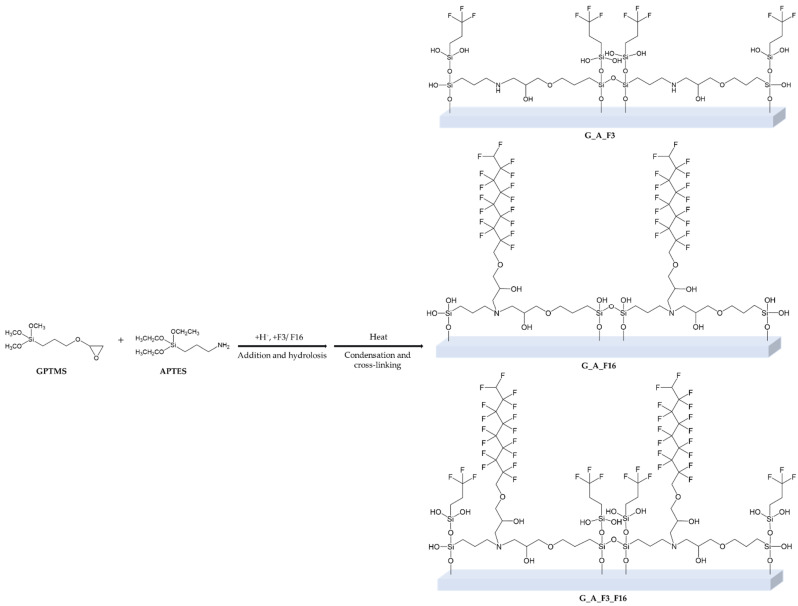
Sol–gel synthesis towards the formation of the three functional G_A_F3, G_A_F16, G_A_F3_F16 coating by reaction of GPTMS/APTES (2:1), and F3 and F16 (overall 5 wt%).

The proper design and development of fluorinated, biocide-free amphiphilic sol–gel-based coating has been run, following the synthetic procedures reported in Figure 4 and Figure 5. 

In particular, the amine group is able to react first of all with one or two epoxy group of the G moiety. Reversely the alkoxysilane ends, after a first acidic hydrolysis step, may statistically bound each other or after the application on a glass surface, in the condensation step may bond stably to the glass or either cross-link each other, giving rise to a diffuse polar polyether amine (PEA) containing polyethylene oxide (PEO) in the G_A sol–gel-based matrix [61].

Moreover, the other three designed sol–gel functional fluorinated mixtures were also prepared by the addition of an overall 5% of F3, F16, or both F3 and F16, i.e., fluorinated functional long or short chain sol–gel precursors. In this way, the overall sol–gel technique will allow the development of a hybrid functional coating, bearing both polar/hydrophilic components related to the amino, ether, and hydroxyl groups, and nonpolar/hydrophobic –CF_3_ (F3) and –C_8_HF_16_ (F16) components, whose FR amphiphilic properties will be tested by mechanical and biological tests.

### 2.1. Characterization of Sol–Gel Coated Glass Slides

#### 2.1.1. FT-IR Analysis

**Figure 6:**
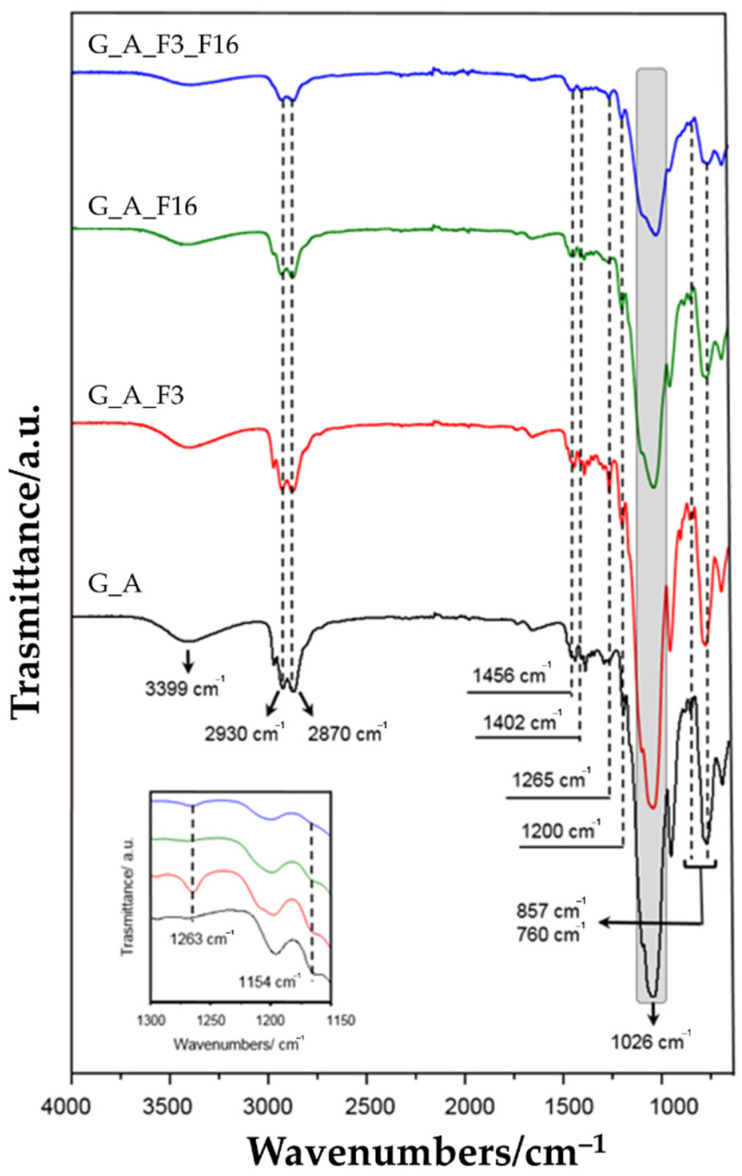
FT-IR spectra of glass slides treated with G_A, G_A F3, G_A F16, and G_A F3_F16 sol.

To investigate the nature of the coatings and to confirm their successful deposition, ATR FT-IR spectra of silane xerogel coatings applied and annealed on glass slides were registered and investigated. The frequencies of major absorption bands are shown in Figure 6 and Table 1, respectively [62].

The nature of the hybrid structure silica precursors based on either epoxy or amino groups is strongly influenced by the epoxy ring opening that can follow different well-known subsequent reaction pathways: (a) hydrolysis with formation of diol; (b) alcoholysis with the formation of ethyl ether terminal groups; (c) consecutive polymerization steps to give oligo- or poly(ethylene)oxide groups or a hybrid 3D network; (d) reaction with primary/secondary amino group, thus transformed in secondary/tertiary. Indeed, during the final thermal curing step, each silanol group obtained by sol–gel precursors hydrolysis, can react with each other to form stable siloxane bonds (Si-O-Si). At the same time, further bonds can be formed by glycidyloxy groups able to react both with themselves and with hydroxyl groups of hydrolyzed precursors. Consequently, the polyaddition reaction of the opened epoxy groups, with the formation of Si-O-C bonds, can increase the flexibility of the network, favoring a great homogeneity between the organic and inorganic components of the so-obtained network.

In the coating (G_A) obtained by the combination of GPTMS and APTES, a broad peak located at 3399 cm^−1^ is dominated by -NH stretching of -NH_2_ group, while some characteristic bands at 2930 cm^−1^ and 2870 cm^−1^ were assigned to C-H symmetric stretching of CH_2_ in the propyl chain present in both precursors. The peak at 1456 cm^−1^ was characterized as a C-H deformation in alkyl chains, while bands at 1026 cm^−1^, 857 cm^−1^ (bending), and 790 cm^−1^ (stretching) were due to Si-O-Si stretching and Si-O-Si bending modes, confirming the formation of an inorganic SiO_x_ matrix. Further bands at 1402 and 760 cm^−1^ were ascribed to the C-N stretching and the N-H out-of-plane bonding, respectively. The coatings realized by adding to the GPTMS/APTES combination two fluorinated precursors, both individually and in combination, show the same hybrid structure. In the coating (G_A) obtained by the combination of GPTMS and APTES, a broad peak located at 3399 cm^−1^ is dominated by -NH stretching of the -NH_2_ group, while some characteristic bands at 2930 cm^−1^ and 2870 cm^−1^ were assigned to C-H symmetric stretching of CH_2_ in the propyl chain presents in both precursors. The peak at 1456 cm^−1^ was characterized as a C-H deformation in alkyl chains, while bands at 1026 cm^−1^, 857 cm^−1^ (bending), and 790 cm^−1^ (stretching) were due to Si-O-Si stretching and Si-O-Si bending modes, confirming the formation of an inorganic SiOx matrix. Further bands at 1402 and 760 cm^−1^ were ascribed to the C-N stretching and the N-H out-of-plane bonding, respectively. The coatings realized by adding to the GPTMS/APTES combination two fluorinated precursors, both individually and in combination, show the same hybrid structure. In addition to the already characterized sol–gel coatings infrared bands, in G_A_F3, G_A_F16, and G_A_F3_F16 samples the presence of fluorine was confirmed by the variation of spectra in the range 1130–1260 cm^−1^, due to the presence of the peaks ascribable to fluorinate groups. Even if partially overlapped by other bands, the shoulder at 1154 and the peak at 1263 cm^−1^ were related to CF stretching in CF2 and CF3 groups. In particular, to confirm this interpretation, it should be noted that the band assigned to CF2 does not appear in the G_A_F3 coating spectrum, since it does not exist in the chain of the 3,3,3-trifluoropropyltrimethoxy-silane precursor.

#### 2.1.2. Morphological and Topography Characterization

The nanoscale morphology of functionalized glass substrates was investigated via atomic force spectroscopy (AFM).

**Figure 7:**
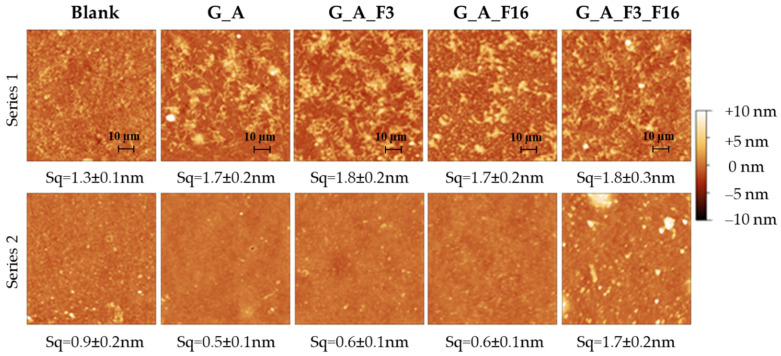
Representative AFM images of bare and functionalized glass substrates (all images are 2 × 2 µm, series 2 differs from series 1 for the presence of an additional applied coating layer).

Bare glass slides were found to have a relatively featureless, homogeneous appearance with a root mean square surface roughness (Sq) of 1.3 ± 0.1 nm. The surface of all G_A-functionalized substrates evidenced numerous ridge-like protrusions with an average length of 62 ± 12 nm (Figure 7). Successive functionalization with F3 and F16 had a marginal impact on the substrates’ surface morphology from both a qualitative and a quantitative point of view, as attested by the fact that the Sq of G_A, G_AF3, G_A F16, and G_A F3_F16 substrates fell within the 1.8 ± 0.3 nm range. Roughness values recorded on the second series of samples were generally lower, with an average Sq of 0.7 ± 0.2, except for sample G_A_F3_F16 which showed a higher Sq of 1.7 ± 0.2. Interestingly, the characteristic ridge-like features of the previous sample set were not visible, while occasional depressions and holes were observed. These observations are compatible with the presence of an additional coating layer of amorphous material, masking the underlying surface, on the second sample set.

**Figure 8:**
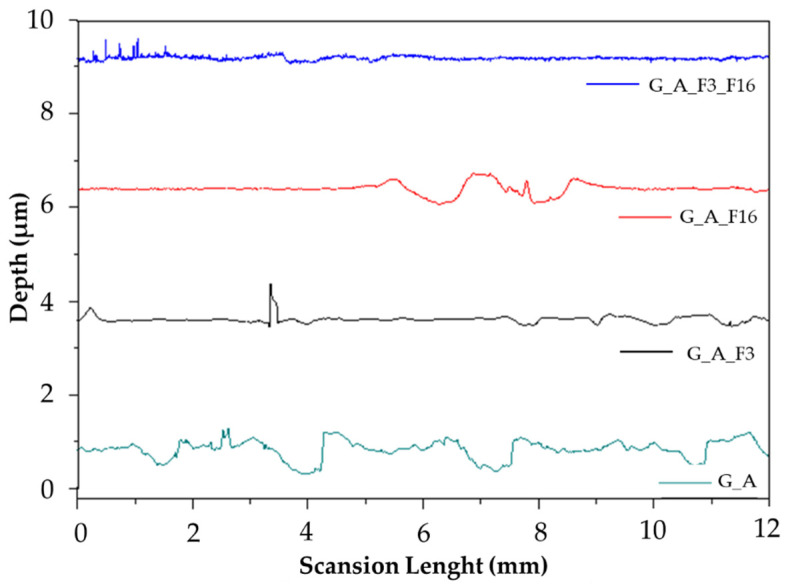
Surface roughness profiles of G_A, G_A_F3, G_A_F16, and G_A_F3_F16 samples.

Figure 8 shows the surface profile of the antifouling coatings, measured with a surface profilometer.

**Figure 9:**
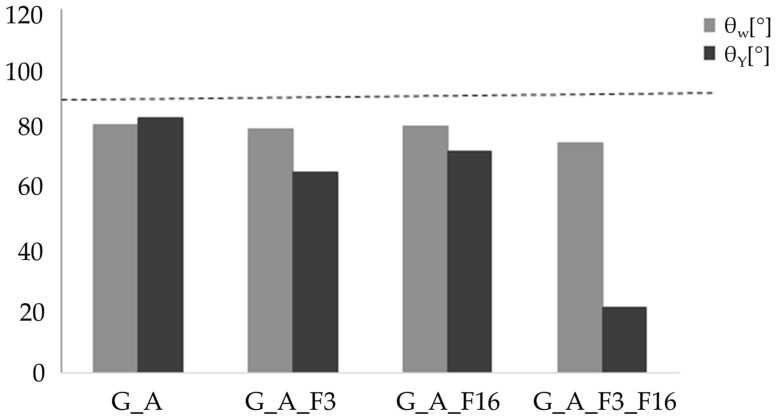
Comparison of contact angles θ_w_ and θ_Y_ of the coatings.

The surface roughness parameters of these coatings (Ra) are listed in Table 2. Functionalization of G and A chains with fluorinated organic compounds should lead to an increase in surface roughness compared to an unmodified matrix, but this roughness decreases in particular for the sample G_A_F3_F16. In fact, all functionalized samples show a decrease in roughness from an Ra value of 0.53 μm for G_A_F16 to an Ra value of 0.26 μm for G_A_F3_F16. In order to fully understand the chemical structure influence of the coating, wettability and surface roughness need to be measured at the same sample spot, and the results evaluated according to Equation (2) to separate the effect of the roughness from the wettability. Figure 9 and Table 2 show the contact angle (θ) values given by Wenzel’s equation and Young’s equation concerning the sol–gel coatings.

Considering that, for hydrophilicity, there is a low contact angle (θ < 90°), while for a hydrophobic situation there is a high contact angle (θ > 90°), the functionalized coatings as such have hydrophilic characteristics, even more than the G_A matrix. The comparison between the contact angles θ_w_ and θ_Y_ of the coatings shows an ideal decrease in the value of the contact angle θ_Y_ with respect to that θ_w_. 

The roughness values of the functional coated surface are in the range expected for the coated glass surface, i.e., 0.53–0.26 μm. On the other hand, the roughness of the coated surface (that may be influenced by the manual “doctor blade” method, as described in the material and method section) affects Young’s contact angle values, since an increase in the roughness will decrease Young’s contact angle value (by applying the Equations (1) and (2) in Section 4.2). Anyway, it is worthwhile to remark that all the developed coatings have both Young and Wenzel contact angles lower than 90 °C, even by employing functional alkoxysilanes featuring fluorinated alkyl chains by increasing length, leading us to conclude that we are dealing with hydrophilic coated surfaces.

### 2.2. Characterization of Sol–Gel Colloidal Solutions 

**Figure 10:**
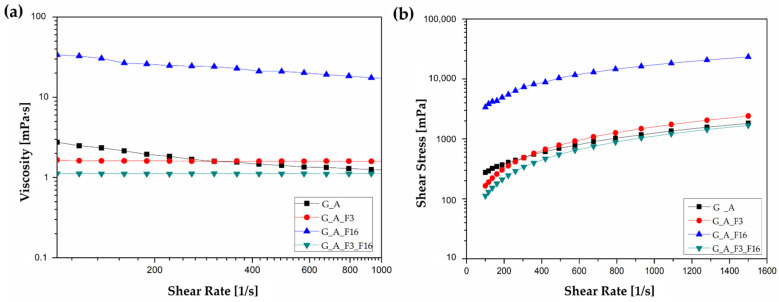
(**a**) Viscosity vs shear rate and (**b**) flow curves of the coatings at room temperature.

In the present study, it was observed that shear-thinning behavior varies as a function of the type of fluorinated compound used for the functionalization. Figure 10a shows the viscosity changes as a function of the shear rate for the different coatings.

Viscosity generally represents the flow resistance of materials based on the interaction between the components. The results revealed that the viscosity was almost dependent on the shear rate, behaving like a Newtonian fluid, and it gradually decreased with increasing shear rate, indicating shear thinning properties [71]. Across the entire measuring range, the coating G_A_F16 exhibited a higher viscosity of η = 22.94 mPa·s within the shear rate range shown (100 s^−1^ to 1000 s^−1^), while the coatings G_A, G_A_F3 and G_A_F3_F16 remains constant, thus showing ideally viscous flow behavior with η = 1.68 mPa·s, η = 1.60 mPa·s and η = 1.12 mPa·s, respectively. The measuring results of viscosity curves are presented as a diagram with shear rate plotted on the x-axis and viscosity plotted on the y-axis; both axes are presented on a logarithmic scale. The relationship between the shear stress and shear rate of the coatings is shown in Figure 10b.

### 2.3. Evaluation of Antifouling Properties and Characterization

The microscopic analysis carried out on untreated (control) and treated glasses showed significant differences in adhered cells between untreated and treated glasses. 

#### 2.3.1. LM and EM 

**Figure 11:**
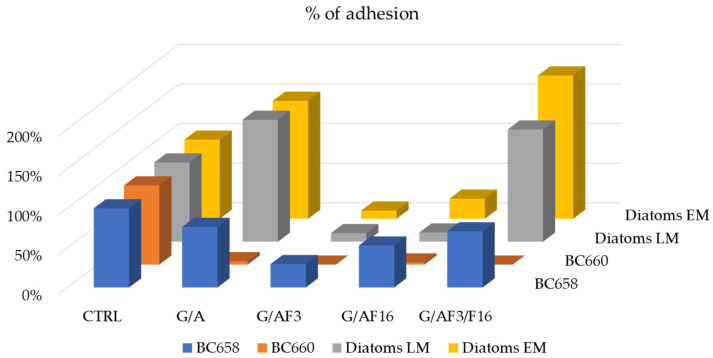
Histogram of the percentage of adhesion on the treated surface of glass slides compared to the adhering cells on the untreated glass slides. For diatoms were reported the results obtained by using two different microscopes (LM and EM).

Figure 11 summarizes all the results of the percentage of adhesion of the three microorganisms tested as compared to the untreated control (=100%). 

**Figure 12:**
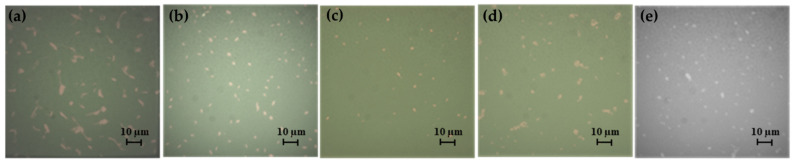
Images of the Gram-negative strain *S. maltophilia* BC 658 observed with an epifluorescence microscope. (**a**) Control 63×; (**b**) G_A 40×; (**c**) G_A_F3 40×; (**d**) G_A_F16 40×; (**e**) G_A_F3_F16 40×.

Each set of experiments carried out with the three microorganisms is shown in the following figures. Figure 12 shows the results obtained with the Gram-negative strain *S. maltophilia* (BC 658). 

All glass slides treated with the four sols determined a significant decrease in adhering cells with respect to the control. G_A covered glass slide reduced the adhered cells number to 76.5% with respect to the control. G_A_F3 and G_A_F16 reduced significantly the adhering cells number, respectively, to 29.5% and 53%. G_A_F3_F16 showed also an FR activity decreasing the adhering cells number to 71% with respect to the control.

G_A_F3 coated glass slide showed the best FR attitude against the Gram-negative strain.

**Figure 13:**
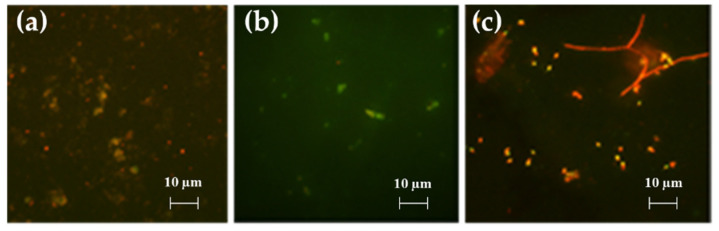
Images of the spore-positive Gram-positive strain *R. aquimaris* BC 660 observed under an epifluorescence microscope. (**a**) control 40×; (**b**) G_A 40×; (**c**) G_A_F16 40×.

Figure 13 shows the results obtained with the strain *Rossellomorea aquimaris* strain BC 660. On the control glass slides, the strain produced abundant biofilm that contributed to the coverage of glass slides, despite the number of cells covering only ~12.5% of the area considered. G_A_F3 and G_A_F3_F16 resulted very effectively against the Gram-positive strain and did not allow bacterial adhesion. A significant decrease in adhering cells was observed. 

**Figure 14:**
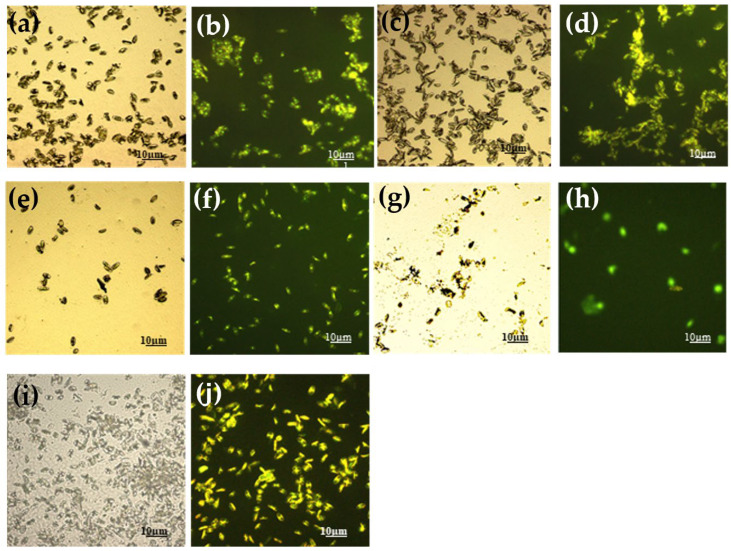
Images of the slides treated and not in contact with *Navicula* sp. obtained with the light microscope and with the epifluorescence microscope 20× magnification. (**a**,**b**) Control; (**c**,**d**) G_A; (**e**,**f**) G_A_F3; (**g**,**h**) G_A_F16; (**i**,**j**) G_A_F3_F16. Note that figures g and h (G_A_F16) showed suffering and ghost cells.

The results obtained with the strain of *Navicula* sp. are shown in Figure 14. 

The coating alone G_A increased the adhesion of the diatom with an increase in the number of 154% (seen under LM) and 149% (for EM) than control. A diminishing number of adhering cells (regarding G_A_F3 covered glass slide) was observed (10.8%) with LM, whereas with a fluorescence microscope number of cells seemed to be similar to the control one (105%). However, in more careful observation of the images, we clearly pointed out that while the cells of the control tend to aggregate into clusters, on the G_A_F3 covered glass slides the cells are spread and not aggregated (Figure 14f). G_A_F3_F16 shows a behavior similar to G_A with a cell number increasing considerably higher with respect to the control (141.7%, 181% in epifluorescence).

#### 2.3.2. Morphological Characterization: Scanning Electron Microscopy (SEM)

**Figure 15:**
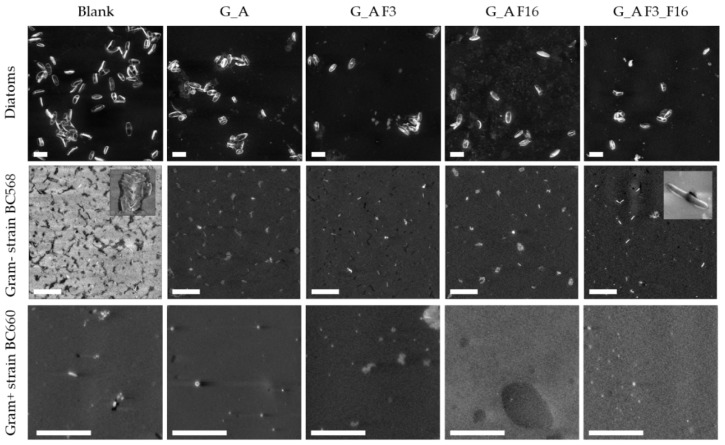
Representative SEM micrographs of bare and functionalized glass substrates after exposition to different microbial suspensions. All scale bars are 20 μm; insets are 20 by 20 μm (on the left, showing clustered bacteria) and 2 by 2 μm (on the right, showing an isolated bacterium).

The influence of different glass functionalization procedures on organism surface adhesion was investigated by SEM imaging (Figure 15). All images showed clearly resolved organisms in different amounts. Individual diatoms were found to measure 80 ± 20 μm^2^ and bacteria 1.4 ± 0.3 μm^2^ when adhered to substrates. The surface densities of organisms and the percentage of surface area covered by them were determined by quantitative SEM image analysis for all substrates.

Bare glass substrates were found to be the most prone to the adhesion of both diatoms (6.9 ± 3.9% surface coverage) and bacteria (1.63 ± 0.29%), whereas functionalized surfaces inhibited their adhesion to different extents. Functionalization with G_A alone was sufficient to drastically drop the surface coverage of diatoms (to 3.3 ± 1.8%) with respect to bare glass substrates, while bacteria were largely unaffected (1.55 ± 0.22%).

Functionalization of G_A treated substrate with F3 and F16, or their combination further depressed diatom surface coverage percentages to, respectively, 2.4 ± 1.0%, 2.1 ± 0.6%, and 1.8 ± 0.1%, thus reaching an almost four-fold decrease in surface adhesion for the G_A_F3_F16 combination. Bacteria were instead mostly influenced by the presence of F3, showing a surface coverage of 0.22 ± 0.02% for the substrate treated with G_A_F3 and of 0.17 ± 0.02% for the G_A_F3_F16 combination. Functionalization with F16 of the G_A treated substrate showed a comparatively milder effect, only reducing bacteria surface coverage to 0.45 ± 0.06%.

The main factor influencing surface coverage of bacteria was observed to be the tendency to form large clusters on certain substrates. 

While the number of adhering bacteria clusters varied only slightly across all substrates, the number of individual bacteria found within those clusters varied considerably. Three substrates showed large clusters (see left insert in Figure 15) of adjoining bacteria containing 10+ individuals (bare glass, G_A, and G_A_F16). Conversely, substrates containing F3 (G_A_F3 and G_A_F3_F16) instead only showed individual, discrete bacteria (see right inset in Figure 15). 

**Figure 16:**
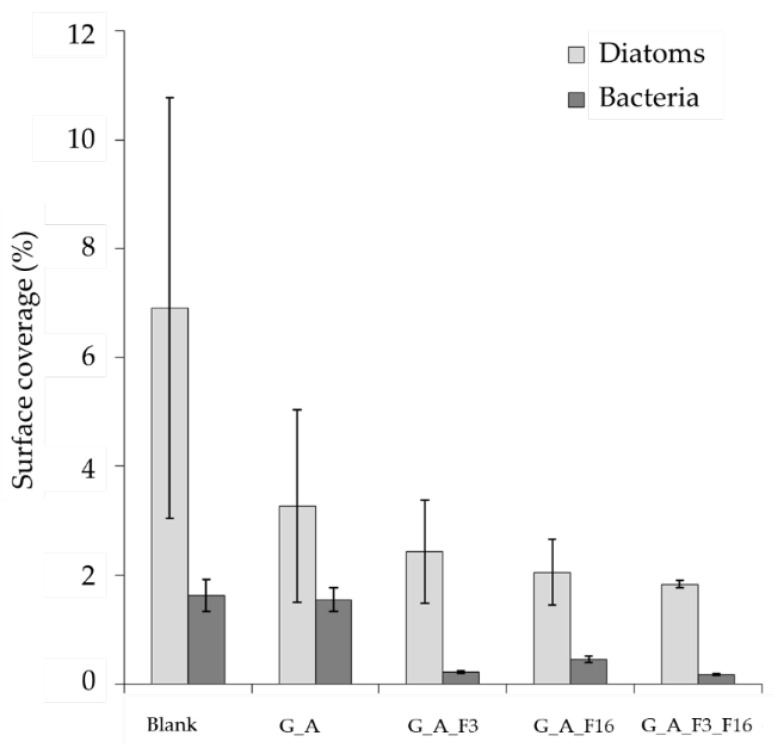
Percentage of area covered by adhered diatoms and Gram-negative bacteria as estimated by SEM imaging.

Figure 16 shows the percentage of surface covered by adhered organisms (i.e., diatoms and the Gram-negative bacteria) as estimated by SEM imaging. 

The sample surfaces with the Gram-positive bacteria are apparently empty due to the biofilm production induced by the Gram-positive strain itself. 

In the SEM images few rod-shaped cells were visible and several spores, for this reason, a statistical analysis of the adhesion of surfaces was not carried out for this strain. 

To support the hypothesis of a biofilm on the surface of the samples treated with the Gram-positive strain, occasionally holes were seen, and in addition, the surface was charged with excess electrons much more than the corresponding series containing the Gram-negative strain.

### 2.4. Evaluation of Toxicity of the Coating against Bacteria and Diatoms

**Figure 17:**
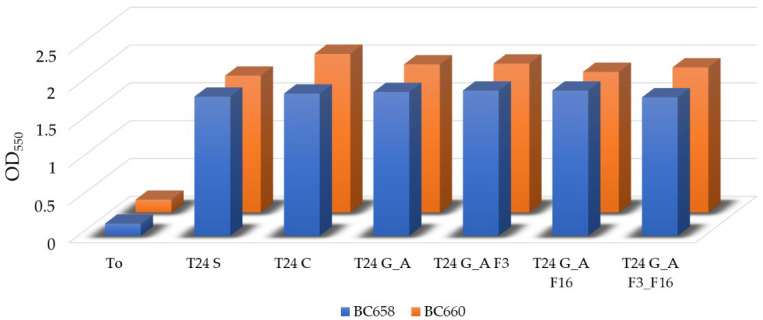
Histogram showing the increase in OD_550_ of the bacterial suspensions before the experiment and after. No significant differences were found between the control suspensions (no glasses, T_24S,_ and untreated glasses T_24C_) and the microbial suspensions coming from the Petri dishes with treated glasses.

No biocide-release effect was observed against both the Gram-positive and Gram-negative bacteria. In fact, the OD_550_ measured before and after the experiment showed an increment of turbidity of 10-fold (from OD_550_ 0.17–0.18 to an average of OD_550_ 1.88 to 1.93, respectively, for BC658 and BC660 (Figure 17). 

**Figure 18:**
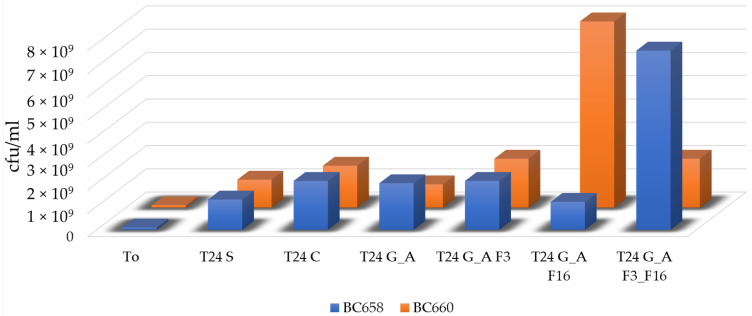
Histogram showing the increase in number of vital bacteria (cfu/mL) after 24 h of incubation in the inoculated liquid medium in presence of untreated and treated glasses. Results were coherent to the OD measurement, except for BC658 in presence of F3/F16 glass slides and for BC660 in the medium in presence of glass slides treated with F16, where a higher number of bacteria was recovered.

**Figure 19:**
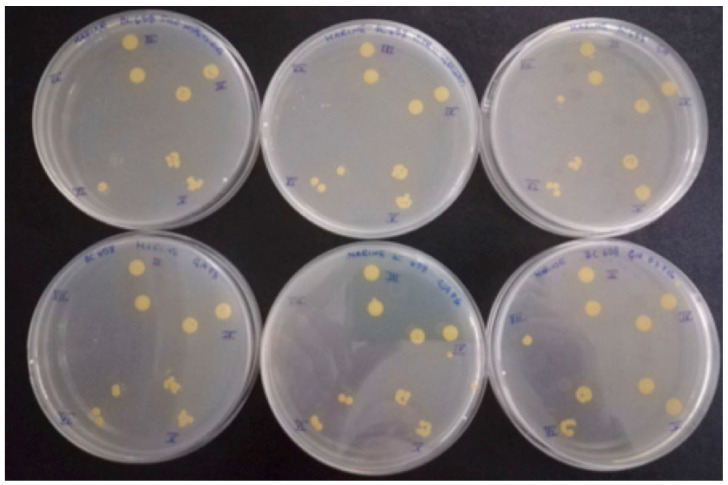
Petri dishes containing marine agar inoculated with the spot technique. Separate colonies were counted only at the higher dilutions.

These values were coherent to the increased number of cell/mL from the initial value of 1 × 10^8^ cell/mL to a value more than 10-fold higher (more than 2 × 10^9^ cell/mL, Figure 18 and Figure 19).

**Figure 20:**
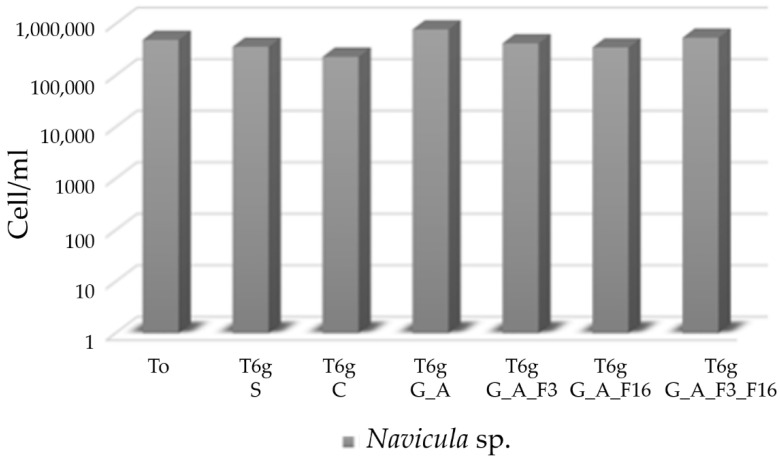
Histogram showing the absence of toxicity against Navicula in contact to untreated and treated glasses as measured through direct count of cells under microscope in Burker counting chamber.

No biocide-release activity was observed against *Navicula* strain as shown in Figure 20. Additionally, in this case, the number of diatom cells after 6 days presented slight differences among suspensions in contact with untreated and treated glasses.

#### Bacterial Adhesion Tests in Simulation Experiment

**Figure 21:**
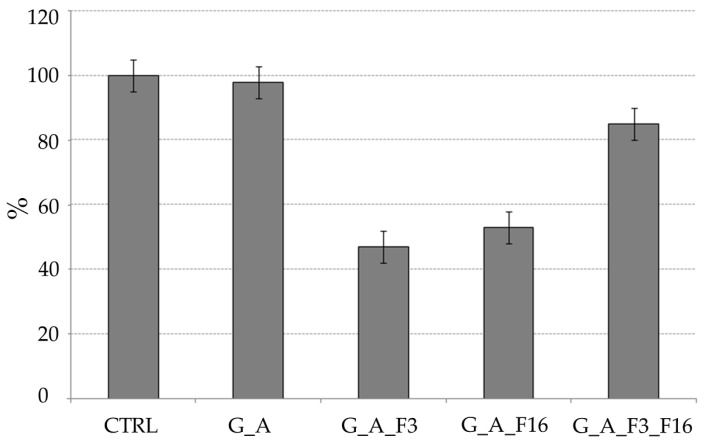
Percentage of bacterial adhesion in contact with untreated (control, CTRL) and treated glasses (G_A, G_A_F3, G_A_F16, and G_A_F3_F16). Area covered by adhering organisms has been estimated by staining with DAPI and counting under epifluorescent microscope.

**Figure 22:**
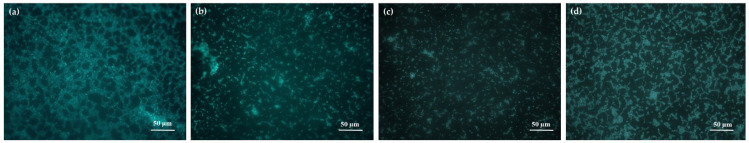
Images of microbial biofilm present on coating surfaces after 60 days of experimentation seen after DAPI staining under fluorescent microscopy (EM); (**a**) G_A, (**b**) G_A_F3, (**c**) G_A_F16, and (**d**) G_A_F3_F16.

Data obtained after 60 days of exposure evidenced, through DAPI staining and fluorescent direct count, are shown in Figure 21 and Figure 22. In untreated glasses (control) and in the G_A system the adhesion of marine bacteria and the consequent biofilm formation is maximal (almost 100%); on the other hand, in alternative systems (treaded glasses) the rate of adhering cells decreased with values of about 47 and 53% for systems G_A_F3 and G_A_F16, respectively. Values of 87% have been registered for G_A_F3_F16 glasses.

Even if all developed fluorinated coated cannot be classified as hydrophobic, as evidenced by wettability tests, all experimental findings led us to conclude that the best FR activity is shown by G_A_F3 coating, or to some extent by the G_A_F3_F16 mixed coating.

**Figure 23:**
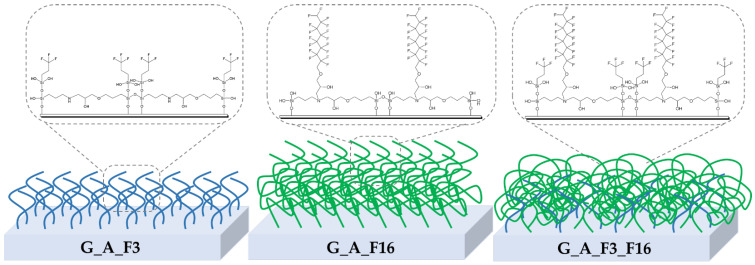
Sketches of F3 and F16 brushes distribution on fluorinated coated glasses.

Rheological analyses run on the mixed G_A_F3_F16 coating show its lowest roughness values among the developed coatings. This behavior may be ascribed to asymmetric hyperbranched developed coated surface in which most probably F3 and F16 collapse each other and flatten after the curing step in a mushroom-like form for the F16 long chain (Figure 23) [72].

As evidenced by SEM analysis, the different extent of diatom/bacteria adhesion inhibition by specific substrates hints at the different adhesion mechanisms employed by the model organisms, which also differ by almost two orders of magnitude in size. 

In this respect, it is not surprising that the change in surface roughness evidenced by AFM imaging has a considerably stronger impact on the larger diatoms with respect to bacteria. As a matter of fact, it is already well-known that a reduction in roughness on the coated or nanocomposite surface can significantly reduce bacterial adhesion [73]. 

In particular, diatoms adhesion differs from bacteria one, not only because of surface roughness, but also for their nano-porous architecture that enhances their adhesion [74] on hydrophobic surfaces and fouling release coatings [75].

**Figure 24:**
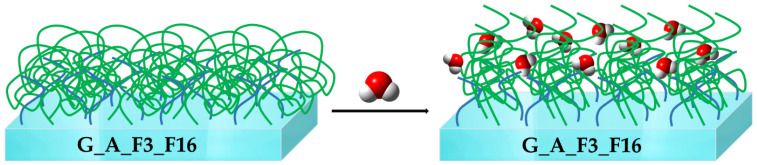
Swelling and elongation phenomena of F16 nonpolar long chain branches taking place at the water/coating interface.

In both cases, the best inhibition of organism adhesion by SEM microscopy was observed on the G_A_F3_F16 substrates. These results led us to conclude that, as shown before [72], in this latter case in the presence of water, there are swelling phenomena taking place at the water/coating interface, leading to an elongation of the F16 nonpolar brushes that re-build the fouling unfavorable and FR asymmetry of the G_A_F3_F16 coating (Figure 24).

## 3. Conclusions

Functional hybrid fluorinated and biocide-free formulations are diffusely employed in order to develop antifouling (AF), fouling release (FR), amphiphilic or hydrophobic marine coatings, based on a physical principle. In particular, this work presents an easy procedure for manufacturing an anti-biofouling/fouling release sol–gel-based polymeric hybrid coating featuring a short- or long-alkyl fluorinated chain. The effective persistence of the amphiphilic character with respect to the replacement of the fluorinate silanes by the alkoxysilanes was ensured by the measurement of the static water contact angle. 

The efficiency of the antifouling/foul release properties was assessed through testing against the adhesion and deposition of selected marine Gram-positive/Gram-negative bacteria and diatoms and by natural marine microbial population experimental microcosm. Chemical–physical and morphological characterization of the coated and uncoated surface before and after microbial adhesion tests have been performed. Microbiological experiments carried out in two different conditions (laboratory and microcosm), were useful to demonstrate that systems F3 and F16, alone or in combination with each other, largely reduce the rate of adhesion through the fouling release mechanism of the newly prepared coatings, while the matrix alone showed behavior similar of the untreated control and in the case of diatoms even a higher rate of cell adhesion. Slight differences were noticed in the different groups of bacteria. In particular, the Gram-positive strain formed on the glass slides control an abundant biofilm that was not observed on the treated glass slides, Further, the products including the system G_A alone did not show toxicity on the tested microorganisms as they were able to grow in the planktonic state. 

All the performed tests indicated the high anti-fouling/fouling release performance of the F3-containing coating (either in combination with F16), in order to prevent microbial biofilm settlement and adhesion.

The results of this work will open the way to further research studies that should be devoted to better sustainable FR coatings, i.e., durable, reliable, stable, easy to be applied on different surfaces (i.e., metals and steel used in ship construction, buildings, cultural heritages) and cost-effective, and last but not least, able to be scaled-up and employed in a large scale.

We firmly believe that this goal may be reached by the use of nanotechnological improved multifunctional and multicomponent sol–gel-based hybrid materials.

## 4. Materials and Methods

### 4.1. Sol–Gel Synthesis and Application

(3-aminopropyl)triethoxysilane (APTES, hereafter A), (3-Glycidyloxypropyl)trimethoxysilane (GPTMS, hereafter G), 3,3,3-trifluoropropyl-trimethoxysilane (F3), glycidyl-2,2,3,3,4,4,5,5,6,6,7,7,8,8,9,9-hexadecafluorononyl ether (F16), absolute Ethanol (HPLC grade) were purchased from Sigma-Aldrich and used without further purification. Before treatment, glass slides were cleaned with a concentrate sulfuric acid/potassium permanganate solution, then washed several times with ultrapure water and dried in an oven at 80 °C for 24 h prior to all experiments.

Four separate solutions (named G_A, G_A_F3, G_A_F16, G_A_F3_F16) all containing GPTMS and APTES in 2:1 = [G]:[A], and F3 and/or F16 at a total 0.5 wt.%, were prepared. In a typical procedure, 2.013 g of GPTMS were mixed with 0.943 g of APTES in 37.48 g of ethanol under stirring. Then 0.2 g of F3 was added to the clear ethanol solution and left at room temperature under stirring for 24 h. The same reactions were also carried out with the other functional alkoxysilane. The squeegee method, also known as “doctor blade”, was chosen as the deposition technique for forming the different coatings. A cylindrical glass rod with a diameter of about 0.5 cm is used to spread about 1 mL of precursor on a glass substrate. The substrate used for coating deposition is always a microscope slide 1 mm thick and 76 × 26 mm in size. Before coating deposition, the slides were pretreated with a piranha solution that could clean the surface of any organic residues and, at the same time, make the glass hydrophilic by hydroxylating the surface. Next, the slides were washed with distilled water and left in the oven to dry for several hours. To ensure greater protection, two layers of each component were deposited, adequately respecting the drying times of the layers. Once the deposition is complete, heat treatment follows to consolidate the gel coating. The heat treatment consists of a controlled heating ramp at 20 °C/min, followed by a holding phase at 180 °C for 10 min.

### 4.2. Characterization

The four sols (G_A, G_A F3, G_A F16, and G_AF3_F16) were fully investigated through FT-IR spectroscopy. Coated glass slides were characterized by scanning electron microscopy (SEM), and atomic force microscopy (AFM). To realize xerogels and investigate their chemical structure by FT-IR spectroscopy, small amounts of each sol were applied on glass slides, the solvent was removed at 80 °C for 2 h, and the thin coatings were cured at 120 °C for 1 h. FT-IR spectra of the combined, hydrolyzed, and cured silane precursors as solid residue removed by glass slides were acquired by means of a Thermo Avatar 370, equipped with attenuated total reflection (ATR) accessory. A diamond crystal was used as an internal reflectance element on the ATR accessory. Spectra were recorded, at room temperature, in the range from 4000 to 650 cm^−1^, with 32 scans and a resolution of 4 cm^−1^. AFM imaging was performed on a Bruker Multimode 8 (Bruker, Billerica, MA, USA) equipped with a Nanoscope V controller and a type J piezoelectric scanner. Micrographs were recorded in PeakForce mode in air using Bruker SNL-A probes (Bruker, Billerica, MA, USA) with a nominal spring constant of 0.4 N/m. Background subtraction and image analysis were performed with Gwyddion v2.48. Surface roughness parameters were calculated as the average value of five distinct 1 μm^2^-sized areas on each sample, using the standard deviation as a measure of the error. The wettability of the sol–gel coating on the microscope slides was evaluated by measuring ten times the height h (mm) and the base diameter d (mm) of 1 μL drop of deionized water on the horizontal surface of the sample, by means of a microlithic syringe (Hamilton, 10 μL). For each material, 10 measurements of the contact angle of deionized water are typically performed, of which the average value with standard deviation was calculated. Wenzel’s contact angle θ_W_ and Young’s contact angle θ_Y_, have been evaluated by the sessile drops method (ASTM D7334) [76,77,78] and they were derived from Equations (1) and (2):

(1) $$\theta_{w}=2\mathrm{arctg}\left(\frac{2h}{d}\right)$$

(2) $$\theta_{Y}=arcos\left(\frac{{\cos\theta }_{w}}{r}\right)$$

where d is the diameter and h the height (both in mm) of the drop, θw is the Wenzel angle apparent dependent on the roughness of the surface, *r* is the surface roughness (Ra), and θ_Y_ is Young’s contact angle of equilibrium on a perfectly smooth surface.

The surface roughness (*Ra*) of the coatings was calculated by using a roughness tester, Surftest SJ-210- Series 178 (Mitutoyo, Milan, Italy) using Equation (3), in which *Ra* is calculated as the arithmetic mean of the absolute values of the deviations of the evaluation profile (*Yi*) from the mean line:

(3) $$Ra=\frac{1}{N}\overset{n}{\underset{i=1}{\sum }}|Yi|$$

The roughness analysis is carried out using a diamond tip that touches the surface of the sample in order to follow its profile. The measurement conditions of the instrument have been set according to the JIS2001 roughness standard: the roughness R profile for compliance, λs = 2.5 μm, λc = 0.8 μm, five sampling lengths and a stylus translation speed of 0.5 mm/s. On average, n. 3 roughness profiles per type of sample were performed and then an average profile was obtained. The viscosities of the coatings were measured by using a modular compact rheometer MCR-502 (RheoCompass Software, Anton Paar Italia S.r.l, Rivoli, Italy). The coatings were tested by using a cone/plate system at a temperature of T = +25 °C. The shear-rate-controlled test was carried out with 18 measuring points using ascending logarithmic steps. The duration for each measuring point was decreased continuously with increasing shear rates, starting at a shear rate of 100 s^−1^ and ending at a shear rate of 1500 s^−1^. Each test was carried out three times.

### 4.3. Evaluation of Antifouling Properties and Toxicity of the Coatings

The evaluation of both antifouling and toxic properties of the proposed coatings was performed in laboratory and microcosm conditions as explained below [79].

#### 4.3.1. Strains and Culture Media

A Gram-negative strain *Stenotrophomonas maltophilia* BC658 (*Xanthomonadaceae*; *Xanthomonadales*; Gamma Proteobacteria) and a Gram-positive spore forming strain *Rossellomorea aquimaris* BC 660 (*Bacillaceae*; *Bacillales*, Bacteria) and a marine diatom strain *Navicula* sp. (*Naviculaceae*; *Naviculales*; Eukarya) were used in all laboratory experiments. All strains (isolated previously from marine habitat) were kept in the strains collection of the Dept. of Chemical, Biological Pharmaceutical and Environmental Sciences (CHIBIOFARAM) of University of Messina, Italy [80,81,82]. Bacterial strains were cultivated and maintained in Tryptone Soy Agar (TSA; Oxoid Limited, Basingstoke, Hampshire, UK) for the AF test they were grown in marine broth MB (Difco) and in marine agar MA (Difco). The diatom *Navicula* sp. strain was kept and grown in F/2 medium [83].

#### 4.3.2. Microbial Suspensions

Bacterial suspensions were prepared from fresh cultures of bacteria grown on TSA Petri dishes (24 h at 28 ± 1 °C) by picking 2–3 colonies and suspending them in phosphate buffer saline (PBS) 0.2 M (130 mM NaCl, 10 mM NaH_2_PO_4_, pH 7.2); bacteria were washed 3 times in PBS 0.2 M, centrifuged at 10,000 rpm (5417R Eppendorf Centrifuge) at 25 °C per 10 min. At the end of this procedure, the resulting pellets were suspended in MB in order to reach an OD550 nm of about 1.0 corresponding to a concentration of about 10^10^ cell/mL. After it, for the AF tests, the final concentration of bacteria was adjusted at about 1 × 10^8^ cells/mL in MB liquid medium. Bacterial suspension density was measured through a Shimadzu UV mini 1240 UV Vis spectrophotometer. For measurement of the number of diatoms, after 7 days of growth in F/2 at 4000 lux and 30 °C, diatoms cells were directly counted through a Bürker chamber and adjusted to a final concentration of about 5 × 10^5^ cells/mL in fresh F/2 medium [84].

#### 4.3.3. Antifouling and Biocide-Release Assessment

Short-term antifouling properties of the different coatings were determined through the evaluation of microorganism’s adhesion to the untreated and treated glasses after 24 h, observed under light microscopy, (LM), epifluorescence microscopy (EM), and scanning electron microscopy (SEM). The toxicity released by the coating to the environment was assessed by the measurement of the suspension density and/or by counting the cells (cfu/mL) before and after the incubations in the liquid medium. Different sets of glasses were used, prepared as described in par. 2.1 (control, G_A, G_A F3, G_A F16, G_A F3_F16 and 4 replicates) were placed in separate sterile glass Petri dishes (Ø 120 mm). Twenty ml of each microbial suspension were then added into the Petri dishes and these latter placed in a horizontal shaker and incubated for 24 h at room temperature set at 25 ± 1 °C for bacteria, and for 6 days in the light at 30 °C for the diatoms. Before and after the incubation time, the density of bacterial suspension inside the Petri dishes was assessed through OD_550_ measurement (as specified in the previous paragraph) and verified by counting the colony forming units (CFU)/mL on marine agar (MA) by using the spots method of inoculation (10 μL of each decimal dilution of the suspension in double). Diatoms number was directly counted as previously described. After the incubation time, glasses were rinsed with sterile PBS, and treated differently: (i) one set was air dried and fixed by placing the glass directly to the Bunsen flame for 30 s observation was carried out in LM after methylene blue staining; (ii) one set was prepared for epifluorescent microscopy and fixed in a 3:1 freshly prepared solution consisting of 3 parts of fixation buffer (4% paraformaldehyde in PBS, pH 7.2) and 1 part of PBS, incubated for 90 min at 4 ± 1 °C. Bacterial cells were then stained with Acridine orange (AO) (0.1 mg/mL) diluted in sterile distilled water 1:2 (*v*/*v*) for 3–4 min and (iii) the last set of glasses was prepared for SEM microscopy by fixing in glutaraldehyde 2.5% in phosphate buffer (PB) 0.1M (pH 7.4) for 6 h at 4 °C and then air dried [85].

#### 4.3.4. Microbial Adhesion Assessment

Adhesion to the untreated and treated glass slides was observed under three different microscopes as it follows:

(a) For LM and EM were performed in a LEICA DM RE equipped with a video camera (LEICA DC 300 F). For each slide, 3 different images of different fields were acquired through the software Leica QWin Color (RGB). After, images were cut to obtain a final dimension of 602 × 602 μm and treated by using the plugin threshold [86] of Image J. 1.52c. In this way, both the number and percentage of the covered surface were determined for each field. The number of cells counted on the untreated glasses was considered as 100% of cells that could adhere to the glass substrate and thus the other cells counting adhering to the different coatings could be more similar or less than 100%, meaning, respectively, an attractive, similar or a repulsive action toward the microorganisms.
(b) SEM images were obtained on a Zeiss EVO LS10 SEM equipped with a LaB6 thermionic electron source and a variable pressure secondary electron detector. No additional treatment was performed prior to SEM observation of samples, which were kept at 3.0 × 10^−1^ Torr during measurements. Images obtained under both microscope observations were analyzed with Image J 1.52c. Surface densities were estimated by counting individual adhered organisms in at least five randomly selected 100 × 100 μm fields on each sample. The average area of adhered diatoms and bacteria was estimated by measuring 20 individual organisms of each type. In each case, standard deviation was used as the error.

### 4.4. Bacterial Adhesion Tests in Microcosm Experiment

**Figure 25:**
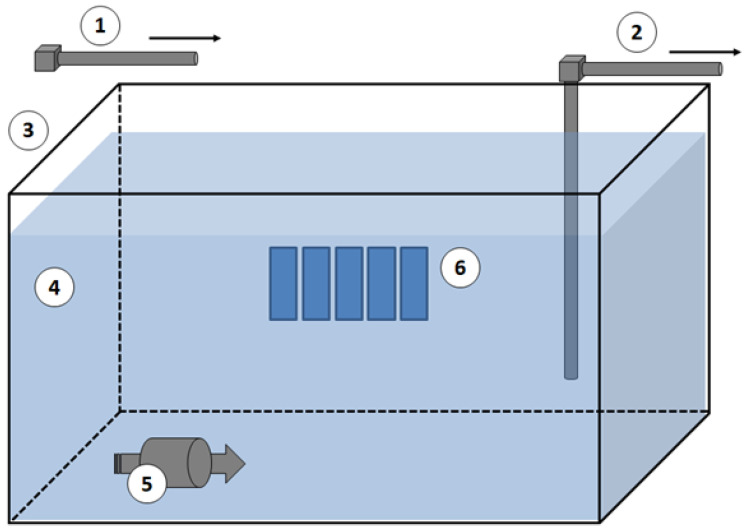
Schematic representation of the microcosm used in the study. Legend: (**➀**) inlet seawater system, (**➁**) outlet “too full” seawater system; (**➂**) experimental tank, (**➃**) seawater, (**➄**) internal pump, and (**➅**) experimental glass.

To test adhesion of natural marine microbial population (in the glass slides covered with different coatings) experimental microcosm was carried out. As reported in Figure 25, the experiment was performed in glass tank (100 × 30 × 40 cm, volume 120 L) filled in continuous (8 L h^−1^) with seawater (salinity 37–38‰) collected directly from the station “Mare Sicilia” (38°12.23′ N, 15°33.10′ E; Messina, Italy) by a pipeline from the sea, in order to ensure approximately two complete water turnover daily. Before introduction to the experimental system, natural seawater was filtered through a 300 μm nylon mesh to remove large metazoans and detritus. To ensure a constant level of water, each microcosm was equipped with a relief valve connected by vertical conduct (PVC-u pn10, 200 mm Ø) placed laterally of the tank to continuously discharge the excess seawater. Water within each microcosm was gently mixed in a continuous mode with a pump (35 L h^−1^) placed close to the entrance of each tank to provide more homogenous conditions within each microcosm. The measurement of pH and temperature, as performed through a multi-parametric probe Waterproof CyberScan PCD 650 (Eutech Instruments, Breda, The Netherlands), and reveal values of temperature of 19.5–20.5 °C (daily temperature fluctuations not exceeded 1 °C) and approximatively constant pH values (around 8). Different sets of glasses (control, G_A, G_A_F3, G_A_F16, G_A_ F3_F16) were inserted inside the experimental microcosm and incubated for 60 days. The glass slides were immersed in a vertical orientation according to the orientation of the other biological tests. The liquid in which the slides were immersed in the previous tests is moved from left to right and right to left (horizontal movement of the shaker), while in the microcosm a similar movement is created by the pump. After the incubation time, the density of bacterial adhesion (biofilm formation) to different support has been evaluated by epifluorescence microscopy (EM). The total bacterial cell counts were performed by DAPI (4′,6-diamidino-2-phenylindole 2HCl, Sigma-Aldrich, Milan, Italy) staining on samples fixed with formaldehyde (2% final concentration), according to Porter and Feig (1980) [87]. Slides were examined by epifluorescence with an Axioplan 2 Imaging (Zeiss) microscope (Carl Zeiss, Thornwood, NY, USA). Results were expressed as number of cells mL^−1^.

## Figures and Tables

**Table 1:** Main vibration modes ascribable to xerogels.

Wavenumbers (cm^−1^)		Reference	Vibrational Modes
***On the glass***	*From literature*		
3399	3450–3261	[63,64]	ν (N-H)
2930–2870	2980–2800	[65,66]	
1456	1450		ν (C-H)
1250	1263	[67]	ν (C-F) in CF_3_
1206	1200	[27,65]	ν (Si-O)
1150	1154	[66,67]	ν (C-F) in CF_2_
1026	1080	[68,69]	(Si-O-Si)
958	950	[69,70]	(Si-OH)
856	816–847	[27,65]	ν (Si-O-Si)
760	786–749	[27,65]	ν_s_ (Si-O-Si)

**Table 2:** Roughness values of Ra and comparison of the contact angles of Wenzel (θ_W_) and Young (θ_Y_) of the coatings.

Name	Ra [μm]	θ_W_ [°]	θ_Y_ [°/μm]
G_A	1.40 ± 0.01	81.84 ± 0.85	84.18 ± 0.85
G_A_F3	0.41 ± 0.03	80.52 ± 0.85	66.31 ± 0.95
G_A_F16	0.53 ± 0.03	81.44 ± 0.85	73.69 ± 0.85
G_A_F3_F16	0.26 ± 0.003	75.80 ± 0.95	21.69 ± 0.95

## Data Availability

Not applicable.
